# Association of *SLIT3* and *ZNF280B* Gene Polymorphisms with Wool Fiber Diameter

**DOI:** 10.3390/ani13223552

**Published:** 2023-11-17

**Authors:** Lin Yue, Zengkui Lu, Tingting Guo, Jianbin Liu, Chao Yuan, Bohui Yang

**Affiliations:** 1 Lanzhou Institute of Husbandry and Pharmaceutical Sciences, Chinese Academy of Agricultural Sciences, Lanzhou 730050, China; 82101215424@caas.cn (L.Y.); liujianbin@caas.cn (J.L.); 2 Sheep Breeding Engineering Technology Research Center of Chinese Academy of Agricultural Sciences, Lanzhou 730050, China; 3 Key Laboratory of Animal Genetics and Breeding on Tibetan Plateau, Ministry of Agriculture and Rural Affairs, Lanzhou 730050, China

**Keywords:** *SLIT3*, *ZNF280B*, fiber diameter, association analysis, Alpine Merino sheep

## Abstract

**Simple Summary:**

Wool is an important raw material in the textile industry. Its fiber fineness is directly related to the wool product quality. Higher quality wool products require finer wool fiber diameters. Therefore, methods for more effectively optimizing the wool fiber diameter have become an important research focus. Single nucleotide polymorphisms (SNPs) are efficient and stable molecular markers which have been extensively used to study wool fiber diameter. The *SLIT3* and *ZNF280B* genes are involved in multiple biological processes and have multiple biological functions. However, the relationship between their polymorphisms and wool fiber diameter remains unreported. This study was conducted to investigate the relationship between different genotypes and wool fiber diameter by typing SNP sites in *SLIT3* and *ZNF280B*. Our results revealed the major genotypes in the *SLIT3* and *ZNF280B* genes which effect wool fiber diameter. These SNP sites may potentially be used as molecular marker sites for optimizing wool fiber diameter.

**Abstract:**

The *SLIT3* gene encodes a secreted protein, and the *ZNF280B* gene is a member of the transcription factor family. Both genes have multiple biological functions. This study was conducted to investigate the association between *SLIT3* and *ZNF280B* gene polymorphisms and wool fiber diameter and to determine potential molecular marker sites for breeding sheep with fine wool. We used Kompetitive Allele-Specific PCR to type the single nucleotide polymorphism (SNP) loci in the *SLIT3* and *ZNF280B* genes within 1081 Alpine Merino sheep and associated these SNPs with wool fiber diameter. The results revealed one SNP in *SLIT3* and *ZNF280B*, which were each related to sheep fiber diameter. The wool fiber diameters of sheep with the CC genotype in *SLIT3* g.478807C>G and AA genotype in *ZNF280B* g.677G>A were the smallest and differed significantly from the diameters of other genotypes (*p* < 0.05). These results suggest potential molecular marker sites for fine-wool sheep breeding.

## 1. Introduction

Wool has been used as a natural fiber in textile production since ancient times. Wool fiber is known for its softness and elasticity, making it a popular choice for clothing and other textiles. Wool products offer many benefits, including fullness, strong hygroscopicity, excellent warmth, and comfort [1]. Wool fiber diameter is one important factor affecting the quality and economic value of wool and is a focus of sheep breeding. Smaller fiber diameters offer better quality wool textiles [2,3]; therefore, fine wool is favored by consumers.

The regulation of wool fiber diameter is a complex process, and according to existing reports, fibroblast growth factor, transforming growth factor, vascular endothelial growth factor, keratin, and other gene families are involved in regulating wool growth and differentiation [4]. The immune response and receptor binding regulatory gene families directly participate in the regulation of wool fiber diameter [5,6]. As a member of the receptor binding regulatory gene family, laminin-1 effects the diameter of hair fibers by participating in differentiating skin hair follicles and wool [7,8]. Fibroblast growth factor 5 has also been shown to synergistically effect wool fiber diameter via single nucleotide polymorphisms (SNP) in the gene [9]. Several genes in the keratin gene family are closely associated with wool fiber diameter, including *KRTAP6-3*, *KRTAP2-1*, *KAP24-1*, and *KAP27-1* [10,11,12,13]. Polymorphisms in these genes are associated with variation in wool fiber diameter. Furthermore, researchers have identified that mutations in the IGFBP3 gene directly influence both wool fiber diameter and length. Notably, the BB genotype appears to reduce wool fiber diameter [14]. 

The *SLIT3* and *ZNF280B* genes used in this study were derived from our previous research results [15,16]. Most previous studies of these genes were conducted on nervous system development and tumorigenesis, while fewer studies focused on wool fiber diameter. According to the available data, *ZNF280* belongs to the zinc finger protein family. As one of the largest transcription factor families in the human genome, this family has many biological functions, including regulating cell growth and development and metabolism [17]. *ZNF280B* is reported to promote prostate cancer cell growth via two main mechanisms. One mechanism enhances the expression of soluble guanylate cyclase (sGCα1), because sGCα1 is an important medium for prostate cancer cell proliferation and represses the p53 tumor suppressor [18,19]. The other mechanism involves binding to the MDM2 promoter to induce its expression, thereby controlling the subcellular localization and stability of p53 to promote cancer cell growth [20]. *SLIT3* is a homolog of SLIT in vertebrates and encodes secretory proteins [21]. *SLIT3* has attracted widespread attention due to its multiple functional properties, including the regulation of axon guidance, cell movement, and angiogenesis. Studies have shown that *SLIT3* plays various roles in different developmental processes and physiological states. For example, in neurodevelopment, *SLIT3* regulates axon guidance and localization by interacting with its receptors, Robo1 and Robo2 [22,23]. In vascular development, *SLIT3* inhibits the proliferation and migration of endothelial cells, thereby effecting angiogenesis and shaping vascular morphology [24,25,26].

Alpine Merino sheep are a new Merino breed bred independently in China, with wool fiber diameters ranging from 19–21.5 μm. These sheep possess the characteristics of both wool and meat production and tolerance to cold and drought. Here, we used Alpine Merino ewes and conducted Kompetitive Allele-Specific PCR (KASP) typing to explore *SLIT3* and *ZNF280B* polymorphisms and their association with wool fiber diameter to provide molecular markers for breeding sheep with fine wool.

## 2. Materials and Methods

### 2.1. Experimental Animals and Traits

The experimental animals came from the sheep breeding technology extension station of Gansu Province, China. We selected experimental animals from a homogeneous population. They were all female, between 4 and 5 years of age, and had similar body condition. We collected 1.5 mL of venous blood from 1081 Alpine Merino sheep with average body condition scores and stored the blood in EDTA-K2 anticoagulant in a 4 °C refrigerator for subsequent KASP typing. Approximately 100–150 g of wool was simultaneously obtained from the posterior edge of the shoulder blade 10 cm above the midline of the body. We collected scapular skin tissue samples, each measuring approximately 1 cm × 1 cm, from 12 live sheep. These samples were placed in tubes and frozen in liquid nitrogen for subsequent RNA extraction and mRNA expression analysis. All animal experiments were performed under the guidance of ethical regulations from the Institutional Animal Care and Use Committee of Lanzhou Institute of Husbandry and Pharmaceutical Science of Chinese Academy of Agricultural Sciences (Approval No. NKMYD201805; Approval Date: 18 October 2018).

### 2.2. Genomic DNA Extraction and Wool Fiber Diameter Detection

DNA was extracted from the 1.5 mL blood samples using a blood genome extraction kit (Genenode Biotechnology Co., Ltd., Wuhan, China). We followed the instructions provided by the kit. The concentration of the DNA was ≥50 ng/μL, and the purity at OD260/280 was 1.7–1.9, which met the genotyping requirements.

At 20 ± 2 °C and a relative humidity of 65 ± 4%, two test samples of 10 g each were taken from the washed and dried wool samples via the multipoint method and dried in an oven at 50 °C for 30 min. After 50 min, a microdrill core sampler was used to cut the fiber segments as test samples, each containing at least 1000 fiber segments. Large plant impurities and overly long fibers were removed from each sample, then all samples were placed into the laser scanner, and the corresponding fiber diameters were read.

### 2.3. Genetic Typing

KASP technology was used for genotyping and was based on the specific matching of the base at the end of the primer to genotype the SNP and detect insertions and deletions. Primer Premier 5.0 software (Premier Biosoft International, San Francisco, CA, USA) was used to design fluorescence quantitative PCR primers. Table 1 shows the primer sequence information. The FS384 realtime fluorescence quantitative PCR system (Changzhou Fusheng Biotechnology Co., Ltd., Changzhou, China) was used for genotyping. The total volume of the real-time fluorescence quantitative PCR was 10 μL:5 μL castPCR Genotyping Master Mix (Genenode Biotech LTCat # 4310A), 0.1 μL upstream typing primer F1, 0.1 μL upstream typing primer F2, 0.3 μL downstream typing primer R, 3.5 μL ddH_2_O, and 1 μL (20 μg/μL) template. The real-time fluorescence quantitative PCR reaction program underwent pre-denaturation at 95 °C for 5 min, denaturation at 95 °C for 20 s, annealing and extension for 1 min for 10 cycles, then denaturation for 20 s, annealing and extension at 57 °C for 1 min for 30–40 cycles, and plate reading at 30 °C for 30 s. If no clear genotyping clusters were obtained, the plate was thermal cycled 2–3 more times, then re-read. The supplementary program underwent denaturation at 95 °C for 20 s, annealing and extension at 57 °C for 1 min, and fluorescence acquisition at 30 °C for 1 min.

### 2.4. SNP Validation

Amplification of the *SLIT3* g.478807C>G and *ZNF280B* g.677G>A was performed to validate the KASP genotyping results. The PCR primers for amplification were designed using the Primer Premier 5.0 software (Premier Biosoft International, San Francisco, CA, USA). The *SLIT3* g.478807C>G is located on chromosome 16 and the *ZNF280B* g.677G>A is located on chromosome 17 of the reference international sheep genome version Oar_v4.0 (accession numbers NC_019473.2 and NC_019474.2, respectively). Amplification of the *SLIT3* g.478807C>G and *ZNF280B* g.677G>A sites in the Alpine Merino sheep was performed using the primer sequences listed in Table 2. The PCR amplification system was composed of 25 µL, including 20 µL Gold Mix (Qinke, Xi’an, China), 1 µL forward primer (Qinke, Xi’an, China), 1 µL reverse primer (Qinke, Xi’an, China), and 3 µL genomic DNA. The amplification of the target loci was performed using the T100 Thermal Cycler PCR instrument (BIORAD, USA) with the following parameters: initial denaturation at 98 °C for 2 min, denaturation at 98 °C for 10 s, annealing at 57 °C for 30 s, extension at 72 °C for 15 s, and a final extension at 72 °C for 5 min. The PCR products were subjected to specific testing using a 1% agarose gel. Samples which showed specific amplification products of the expected length were sent to Qinke Zexi Biotechnology Co., Ltd. (Xi’an, China) for Sanger sequencing. The sequencing results were analyzed using the seqman subroutine (version 7.1.0, DNASTAR Inc., Madison, WI, USA) in the DNAstar software.

### 2.5. Real-Time Quantitative PCR

We extracted RNA from the skin tissue using trizol reagent and reverse transcribed the skin tissue RNA into cDNA for quantitative expression analysis according to the instructions of the reverse transcription kit (TransGen Biotech, Beijing, China). Primers for qPCR were designed based on the sheep *SLIT3* and *ZNF280B* gene sequences (GenBank accession numbers: NC_019473.2, NC_019474.2) (Table 3), with *GAPDH* serving as the reference gene. TransStart Top Green qPCR SuperMix (TransGen Biotech, Beijing, China) was run on a Light Cycler 96 (Roche, Basel, Switzerland). The reaction mixture consisted of 0.4 μL each of the forward and reverse primers, 10 μL of 2× T TransStart^®^ Top Green qPCR SuperMix, 8.2 μL of ddH_2_O, and 1 μL of cDNA. The RT-QPCR parameters were set as follows: predenaturation at 94 °C for 30 s; amplification at 94 °C for 5 s, 58 °C for 15 s, and 72 °C for 10 s, for 45 cycles; and the melting curve was drawn at 95 °C for 5 s, from 65 °C to 95 °C, +0.5 °C, in 5 s. The data were normalized and the relative expression of mRNA was calculated by the 2^−ΔΔCt^ method [27].

### 2.6. Statistical Analysis

Homozygosity (HO) was calculated online using GDICALL (http://www.msrcall.com/gdicall.aspx; accessed on 20 June 2023). Heterozygosity (HE), the effective number of alleles (NE), polymorphism information content (PIC), and the genotype and allele frequencies of the two sites were counted, and Chi-square and Hardy–Weinberg *p*-values were calculated. 

We used one-way analysis of variance (ANOVA) in IBM SPSS Statistics 22 (IBM, Armonk, NY, USA) to investigate the relationships between the polymorphisms of *SLIT3* and *ZNF280B* and wool fiber diameter. One-way ANOVA in IBM SPSS Statistics 22 was used to compare the differences in gene expression among individuals with different genotypes, and the results are expressed as mean ± standard error. To analyze the factors influencing wool fiber diameter, including age and genetic effects, we applied a general linear model and appropriately simplified the model based on the current context. The finished model employed Equation (1), in which *Yijk* is the record of individual phenotypes, *µ* is population mean, *Xi* is the effect due to age, *SNPj* is the fixed effect of the genotype category of the locus, and *eijk* is random error. The differences between means were tested using Duncan’s multiple comparison test, and the results are expressed as the mean ± standard error. The Cor function was used to estimate the correlation between gene expression and wool fiber diameter:

*Yijk* = *µ* + *Xi* + *SNPj* + *eijk* (1)

## 3. Results

### 3.1. Genotyping Results for SLIT3 and ZNF280B and Population Index Analysis of the Loci in Alpine Merino Sheep

**Figure 1:**
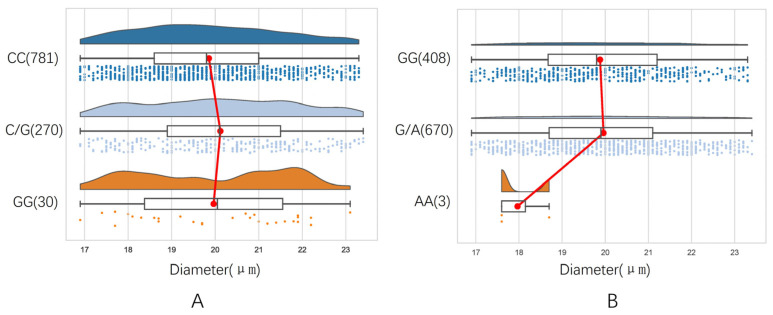
Distribution of fiber diameter in different genotypes. (**A**) *SLIT3* g.478807C>G, (**B**) *ZNF280B* g.677G>A.

Table 4 shows estimates of the genetic parameters of the *SLIT3* and *ZNF280B* loci. Figure 1 shows the genotypic distribution of the two genes. *SLIT3* g.478807C>G was in Hardy–Weinberg equilibrium in the population. The CC, CG, and GG genotypic frequencies in *SLIT3* g.478807C>G were 0.722, 0.250, and 0.028, respectively. The CC was the most common genotype in this population. The C and G allelic frequencies were 0.847 and 0.153, respectively, which indicates a high percentage of unmutated alleles in this population, suggesting that this group contained only small-scale mutations. The HE and NE were 0.259 and 1.349, respectively, and the PIC was 0.225, showing low polymorphism [28].

The GA genotypic frequencies in *ZNF280B* g.677G>A was 0.620, which was the dominant genotype in this population. The G and A allelic frequencies were 0.687 and 0.313, respectively, indicating a high percentage of unmutated alleles, HE and NE, were 0.430 and 1.754, respectively. The χ^2^ value was too large and the *p*-value was less than 0.05. Therefore *ZNF280B* g.677G>A is not in Hardy–Weinberg equilibrium, The PIC was 0.337, indicating moderate polymorphism and showing that *ZNF280B* g.677G>A exhibited relatively abundant polymorphism in this population.

The fiber diameter distribution corresponding to each genotype in *SLIT3* g.478807C>G fluctuated greatly, especially the fiber diameter corresponding to the GG genotype (Figure 1A). In ZNF280B g.677G>A, the distribution of the fiber diameter corresponding to GG and GA genotypes was relatively uniform. The number of individuals with the AA genotype was small, and the distribution of the corresponding fiber diameter varied greatly (Figure 1B).

### 3.2. Association Analysis between ZNF280B and SLIT3 Genotypes and Wool Fiber Diameter of Alpine Merino Sheep

The results of the association analysis between genotypes of *ZNF280B* and *SLIT3* and wool fiber diameter are shown in Table 5. *SLIT3* g.478807C>G was associated with wool fiber diameter. The wool fiber diameter of the CC genotype was lower than that of the CG genotype (*p* < 0.05), indicating that sheep with the wild type at this locus had smaller fiber diameter than that of the sheep with the mutant genotype and that the wild-type sheep had better wool quality. In the case of *ZNF280B* 237 g.677G>A, we observed that the wool fiber diameter was higher in the GG and GA genotypes compared to the AA genotype. The difference in wool fiber diameter between the AA genotype and the other two genotypes was statistically significant (*p* < 0.05). Furthermore, mutants exhibited finer wool compared to the wild-type sheep. 

### 3.3. Expression Analysis of SLIT3 and ZNF280B in Skin Tissue

**Figure 2:**
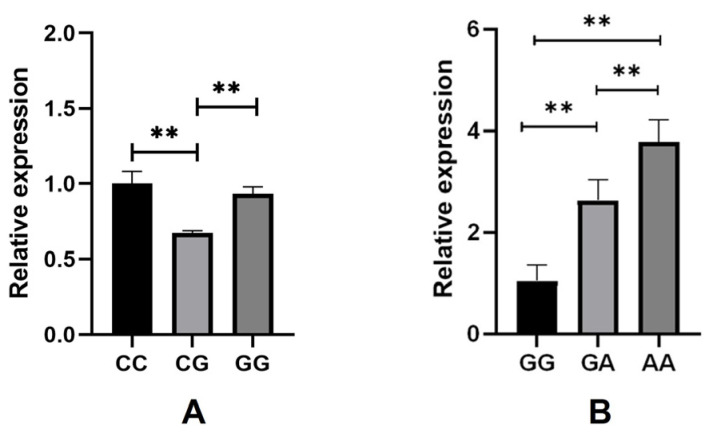
The expression of *SLIT3* and *ZNF280B* in skin tissues of individuals with different genotypes. (**A**) *SLIT3*, (**B**) *ZNF280B*. ** Indicates a highly significant difference (*p* < 0.01).

**Figure 3:**
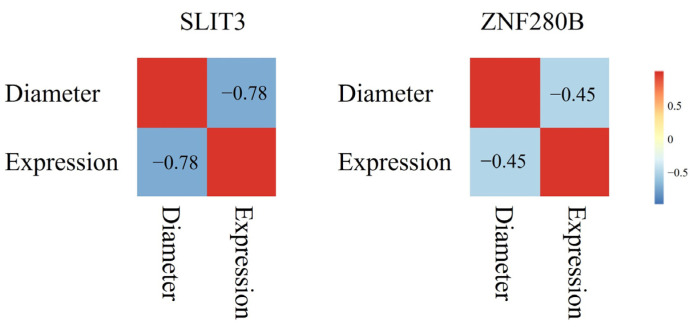
The correlation between gene expression and wool fiber diameter.

We conducted a study on the expression of *SLIT3* and *ZNF280B* genes in skin tissues of different genotypes. As shown in Figure 2, the expression of the *SLIT3* gene in CC genotype and GG genotype individuals was similar. The expression level in CG genotype individuals was less, and the expression level of CC genotype individuals and GG genotype individuals was highly significantly different from that of CG genotype individuals (*p* < 0.01). The expression level of the *ZNF280B* gene was the highest in AA genotype individuals and the lowest in GG genotype individuals, and the expression levels of *ZNF280B* in these three genotypes were highly significantly different from each other (*p* < 0.01). In addition, we analyzed the relationship between gene expression and wool fiber diameter. As shown in Figure 3, the correlation coefficient between the expression level of *SLIT3* and wool fiber diameter was −0.78, a strong negative correlation. The correlation coefficient between the expression of the *ZNF280B* gene and wool fiber diameter was −0.45.

## 4. Discussion

Wool is an important raw material in the textile industry; its fiber diameter is particularly important, and high-quality wool products have high fiber diameter requirements. Therefore, effective methods for improving wool fiber diameter have become an important research topic. SNPs are an important basis for studying genetic variations among livestock and poultry. SNPs show high reliability and are widely distributed and easily analyzed. They play important roles in analyzing the genetic bases of important economic traits in livestock and poultry [29,30].

Here, we studied two SNP loci in *SLIT3* and *ZNF280B*. The *ZNF280B* g.677G>A is located in the coding region of the *ZNF280B* gene. This region has an impact on gene expression and function, thus exerting a more direct influence on the phenotype. As a transcription factor, *ZNF280B* is involved in many biological processes and is naturally the most likely to be involved in regulating hair follicles [18]. We speculate that *ZNF280B* g.677G>A may be involved in the regulation of wool fiber diameter by affecting the expression of *ZNF280B*. The *SLIT3* gene has not been implicated in previous studies in relation to wool fiber diameter. While *SLIT3* g.478807C>G is located within an intronic of *SLIT3*, it is important to note that SNP sites in introns remain valuable for study. These SNPs located in intronic regions cause shear abnormalities by altering the structure of the shear site, which, in turn, leads to abnormal protein structure and function [31]. Furthermore, multiple SNP sites in the intron region of *FGF5* are reported to be significantly associated with wool fiber diameter, and a site in the seventh intron region of *CRY1* effects racing performance in pigeons [9,32]. In addition, our study revealed that the fiber diameter varies with *SLIT3* expression. Gene expression was highest in sheep with the CC genotype, while the fiber diameter was smallest in individuals with this genotype. Expression of the *SLIT3* gene was negatively correlated with wool fiber diameter, that is to say, the higher the expression of *SLIT3*, the finer the wool. A similar phenomenon was shown in individuals with the CG and GG genotypes, that is, the higher the expression of the *SLIT3* gene, the smaller the diameter of the wool fiber. The expression of the *ZNF280B* gene follows a similar pattern. Individuals with the GG genotype exhibit comparatively lower expression than those with GA and AA genotypes, with the corresponding fiber diameter being the largest among these three genotypes. Of course, fluorescence quantification alone cannot fully prove that *SLIT3* and *ZNF280* genes are closely related to fiber diameter, and further research is needed.

We found differential expression of *SLIT3* g.478807C>G and *ZNF280B* g.677G>A in the population of Alpine Merino sheep. *ZNF280B* g.677G>A clearly deviates from Hardy–Weinberg equilibrium in this population. This could be attributed to various factors such as sampling bias, genotyping errors, natural selection, or human choice. In our study, we employed the KASP method, which is specifically designed for the genotyping of large samples at specific SNP loci, a common practice in many studies [33,34]. Therefore, genotyping is unlikely to be the cause of this phenomenon. Inbreeding or outbreeding would not typically result in such significant deviations. Sampling bias is also unlikely, as we did not intentionally select specific samples during the sampling process. Regarding natural selection, it is not likely to be a major factor, as the sheep population is already well adapted to the current environment. After excluding numerous possibilities, we believe that human intervention is the most likely cause of the deviation from Hardy–Weinberg equilibrium. However, this hypothesis requires extensive verification. Additionally, we did not consider DNA mutations or recombination in our study. If we had used sequencing methods for genotyping, it could have helped explain this issue. Considering all of these factors, we believe that human choice is the most plausible explanation. 

The current research on SNP loci associated with wool fiber diameter is not satisfactory, as the majority of the identified SNP loci belong to the KAP and KRTAP families, although KAP and KRTAP play crucial roles in hair follicle development and wool composition [35,36]. However, the regulation of wool fiber diameter is a complex process and cannot be solely determined by KAP and KRTAP families. In this study, building upon previous GWAS studies, we investigated the polymorphisms of *SLIT3* and *ZNF280B* and identified the SNP loci associated with wool fiber diameter. Although the two SNPs we found have a limited effect on wool fiber diameter, this provides more options for further research beyond the genes of the KAP and KRTAP families.

There are hundreds of millions of known SNPs, and the number is increasing, but only a few of these SNPs have been found to be associated with certain biological functions. The two SNPs in this study have been annotated in the sheep genome, but the biological functions of these two sites have not been discussed in previous reports. However, this study only explored two of the many SNPs and did not further explore whether there are sites with similar biological functions near these two sites, because the sites with closer distance generally have strong linkage relationships. If it can be found, multiple sites with linkage relationships are often more meaningful than a single site, especially for quantitative traits such as wool fiber diameter. The more relevant SNPs that are available, the more useful they are in marker-assisted selection.

## 5. Conclusions

In summary, we explored *SLIT3* and *ZNF280B* gene polymorphisms and analyzed the relationship between these polymorphisms and wool fiber diameter. The results showed that *SLIT3* g.478807C>G and *ZNF280B* g.677G>A were associated with wool fiber diameter. Our results may contribute to the development of molecular marker sites for optimizing wool fiber diameter.

## Figures and Tables

**Table 1:** qPCR primer information.

SNPs	Primer Sequence (5′-3′)	Reaction Component
	F1: GAAGGTGACCAAGTTCATGCTCAGGAAAGCAGGGAAGCG	A-FAM
*SLIT3* g.478807C>G	F2: GAAGGTCGGAGTCAACGGATTCAGGAAAGCAGGGAAGCC	G-HEX
(chr16:945395)	R: GCTGGCTTTCTCTGATTCAGC	Common
	F1: GAAGGTGACCAAGTTCATGCTGCATACATAGGGCATTTCGC	G-FAM
*ZNF280B* g.677G>A	F2: GAAGGTCGGAGTCAACGGATTGGCATACATAGGGCATTTCGT	A-HEX
(chr17:70269373)	R: CTGCGGTCTGTAAAATCTGTGAA	Common

**Table 2:** *SLIT3* g.478807C>G and ZNF280 B g.677G>A site amplification primer sequence.

SNPs	Primer Sequence (5′-3′)	Product Size	Position
*SLIT3* g.478807C>G	F: TTTCCCAGCAGGAGTGTCTA	663 bp	Intron
	R: GCCTTCTTGTTTCCGTTTCA		
*ZNF280B* g.677G>A	F: TGGAACACTGGTGAAAACTC	280 bp	Exon
	R: CCCTCTGCGGTCTGTAAAAT		

**Table 3:** Primer sequence of RT-qPCR.

Gene	Primer Sequence (5′-3′)	Product Size
*SLIT3*	F: CCTGGAGTGAAGAAGTGGGA	320 bp
	R: CATGTTCATCCTTTTGTTTT	
*ZNF280B*	F: CAAATTCTTAGTGTTTCCATG	247 bp
	R: CCAGTTTCCCACCCCATTCC	
*GAPDH*	F: AGAAGGCTGGGGCTCATTTG	258 bp
	R: AGGGGCCATCCACAGTCTTC	

**Table 4:** Variation information and diversity parameters of *SLIT3* and *ZNF280B* loci.

SNPs	Genotypic Frequencies			Allelic Frequencies		He	Ne	PIC	*χ* ^2^
*SLIT3* g.478807C>G	CC(781)	CG(270)	GG(30)	C	G	0.259	1.349	0.225	1.282
	0.722	0.250	0.028	0.847	0.153				
*ZNF280B* g.677G>A	GG(408)	GA(670)	AA(3)	G	A	0.430	1.754	0.337	211.188 *
	0.377	0.620	0.003	0.687	0.313				

Note: He: heterozygosity; Ne: effective number of alleles; PIC: polymorphism information content. PIC < 0.25, low polymorphism; 0.25 < PIC < 0.5, moderate polymorphism; PIC > 0.5, high polymorphism; χ^2^ * means *p*-value < 0.05, not in Hardy–Weinberg equilibrium.

**Table 5:** Association analysis between *SLIT3* g.478807C>G, *ZNF280B* g.677G>A, and wool fiber diameter (MEAN ± SE).

SNPs	Genotype	Wool Fiber Diameter (μm)	F
*SLIT3* g.478807C>G	CC	19.8 ± 1.6 a	2.613
	CG	20.1 ± 1.7 b	
	GG	19.9 ± 1.7 ab	
*ZNF280B* g.677G>A	GG	19.9 ± 1.6 a	2.451
	GA	19. 8 ± 1.6 a	
	AA	17.9 ± 0.6 b	

Note: Within a given SNP, different lowercase letters showed significant differences (*p* < 0.05) between the genotypes, F-values indicate differences in means between genotypes within a SNP.

## Data Availability

The data presented in this study are available on request from the corresponding author.
